# Fungal diversity of “solom” a Ghanaian traditional beverage of millet (*Pennisetum glaucum*)

**DOI:** 10.1002/fsn3.2045

**Published:** 2020-11-29

**Authors:** Nii Korley Kortei, Prince Asiedu, Theophilus Annan, John Gameli Deku, Adjoa Agyemang Boakye

**Affiliations:** ^1^ Department of Nutrition and Dietetics School of Allied Health Sciences University of Health and Allied Sciences Ho Ghana; ^2^ Food Microbiology Division Food Research Institute‐Council for Scientific and Industrial Research Accra Ghana; ^3^ Department of Medical Laboratory Sciences School of Allied Health Sciences University of Health and Allied Sciences Ho Ghana; ^4^ Department of Biomedical Sciences School of Basic and Biomedical Sciences University of Health and Allied Sciences Ho Ghana

**Keywords:** ‘solom’, beverages, cereals, fungi, Ghana, millet, pH

## Abstract

The association of cereals with fungi cannot be disregarded as their manifestation in our foods poses serious health risks. The aim of this study was to investigate the mycofloral (fungal) and chemical (pH) qualities of the “solom” (beverage of millet) available for consumption from their respective sales points in Ho. “Solom” a cereal beverage of millet was sampled from ten (10) different locations in the Ho Municipality of Ghana and evaluated for their pH, fungal counts, and species diversity. Mycological analyses were done on Oxytetracycline Glucose Yeast Extract (OGYE) and Dichloran Rose Bengal Chloramphenicol (DRBC) media from three (3) points per location using serial dilution. A total of fourteen (14) fungal species belonging to eight (8) genera were isolated on both media; *Aspergillus (A. niger, A. flavus, A. fumigatus, A. parasiticus, A. alutaceaus, A. terreus), Rhizopus (R. stolonifer), Mucor (M. racemosus), Fusarium (F. oxysporum), Penicillium (P. digitatum, P. verucosum), Cladosporium (C. cladosporoides), Curvularia (C. lunata),* and *Rhodotorula* sp. were recorded. Fungal counts on both media ranged between 1.68 ± 0.8 and 4.11 ± 0.9 log_10_ CFU/ml. There were statistically significant (*p* < .05) differences observed in the samples from different locations. The values of pH recorded were in the range of 3.03 ± 0.09–4.03 ± 0.23 and showed no significant differences (*p* > .05) among them. All samples were found to be in the acceptable range of values prescribed by the International Commission for Microbiological Specification of Foods (ICMSF, 1998). Good Manufacturing Practices (GMP) and Good Hygiene Practices (GHP) should be employed to enhance food safety.

## Graphical abstract

Fungi associated with solom a cereal beverage of millet.

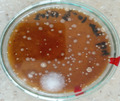

## 1 INTRODUCTION

Most African countries place a premium on the production and consumption of cereal‐based traditionally fermented beverages which are largely of nutritional, medicinal, socioeconomic, recreational, and cultural importance (Aka et al., 2014; Amadou et al., 2011). Tropical cereals such as maize (*Zea mays*), pearl millet (*Pennisetum glaucum*), finger millet (*Eleusine coracana*), sorghum (*Sorghum bicolor*), and fonio (*Digitaria exilis*) are often used singly or in combination in the preparation of an assortment of traditional fermented beverages (Aka et al., 2014; Ezekiel et al., 2015, 2019; Khaneghah et al., 2019; Misihairabgwi et al., 2018). Examples of cereal‐based beverages from Africa are pito (West Africa), brukutu (Nigeria, Ghana), asana and ice‐kenkey (Ghana), tchapalo (Cote d'ivoire), Kununzaaki (Nigeria, Cameroun), akamu (Nigeria), oshinkundu (Namibia), chibwantu and munkoyo (Zambia), and mqomboti/umqumbothi (South Africa) beverages just to mention a few.

**Plate 1:**
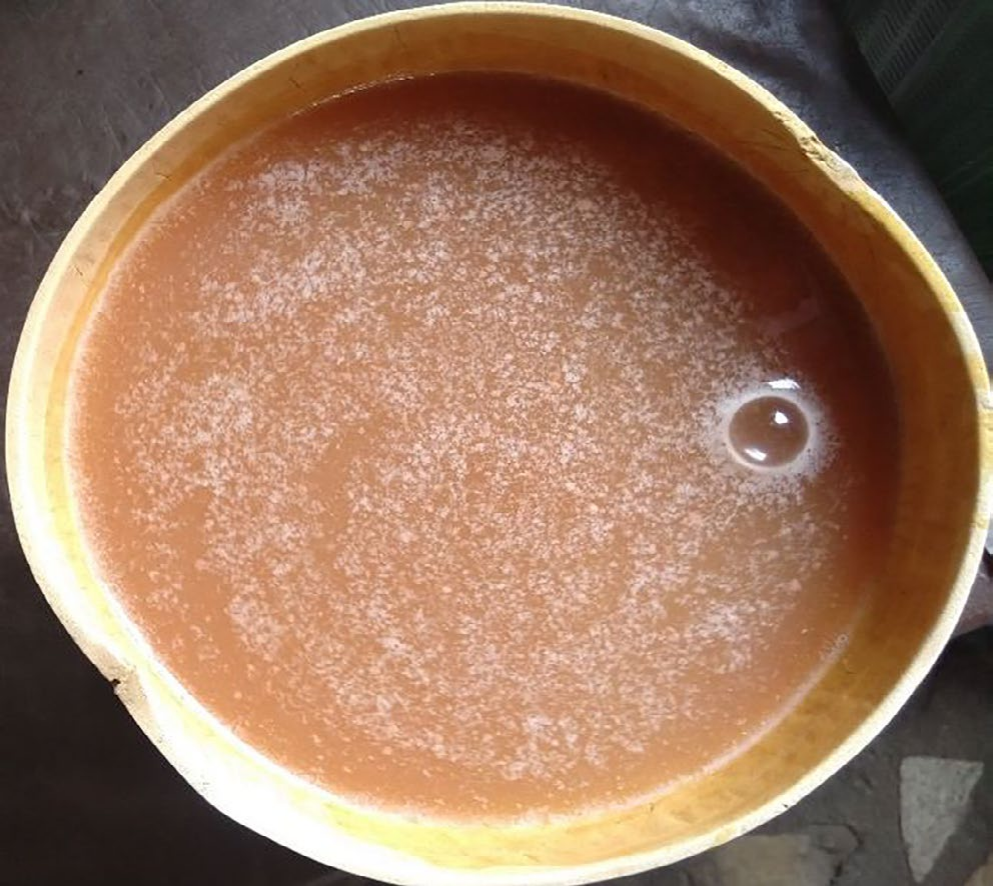
Solom fetched in a calabash ready for drinking as sold on the markets

“Solom” a relatively lesser‐known cereal beverage obtained from millet is consumed in West Africa particularly in Ghana, Togo, and some parts of Benin. It is an opaque (Plate 1) reddish‐brown drink usually served chilled (with ice blocks). It is used for refreshment in both the rural and urban settlements. Culturally, the serving of solom like many other cereal‐based beverages in Africa to visitors is considered a gesture of welcome and hospitality, and the beverage is served as part of the traditional initiation of young girls into womanhood (Misihairabgwi & Cheikhyoussef, 2017). “Solom,” according to oral tradition, is served at traditional weddings and other important ceremonies as well as at daily social interactions (Embashu et al., 2013). The main ingredients for “solom” production are pearl millet meal, sorghum, or maize malt and water. The bran of millet is optionally added. Fermentation is achieved via chance inoculation, under uncontrolled environmental conditions, and the peculiar nutritional and sensory properties of “solom” derived mainly from the ingredients, with variable qualities which depend largely on the skills of the households. In some cases, back slopping is conducted by the local producers to enhance fermentation and product quality.

Fungal contamination of seeds, grains, and feedstuff is a chronic problem in the developing countries because most of the tropical hot and humid climates support the growth of these fungi in the field and storage systems. The presence and growth of fungi mostly cause spoilage of food quality and quantity‐wise. These agricultural raw ingredients have been implicated in the transfer of fungal contaminants to processed foods (Candlish et al., 2001; Rasooli & Abyaneh, 2004).

The occurrence of some genera of fungi in our body system (mycosis) may be detrimental subsequently, certain mycotoxins (mycotoxicosis) produced have carcinogenic, mutagenic, teratogenic, and immunosuppressive implications on humans and animals alike. This is of major concern in evaluating the safety of the final product solom (Mastanjević et al., 2018). Despite the numerous reports on the spectrum and quantities of fungi and their metabolites in cereals used in the preparation and processing of traditional beverages, few studies have analyzed the transfer rate of fungal spores from ingredients to beverages, their concentrations in the beverages, and exposure rates for consumers (Ezekiel et al., 2019).

On another hand, filamentous molds are also responsible for the quality of alcoholic beverages including nutritional values and organoleptic properties such as flavor, taste, and color (Tamang et al., 2016). It is noteworthy that no strict process control is imposed on traditional brewing, fermentation, or storage of raw cereal grains in Ghana. Poorly enforced regulations targeted at ensuring the microbiological safety levels in African traditional fermented beverages exist, despite the high fungal contamination levels in their raw materials and the high consumption levels of the beverages (Ezekiel et al., 2019). Fungal detection, quantification, and determination of fate during processing are therefore imperious in the cereals and traditionally fermented beverages.

Although scanty data exist (Aboagye et al., 2020; Minamor, Mensah, et al., 2017) on the fungal diversity of cereal‐based drinks in Ghana, the ability of fungi to grow on some cereals has been demonstrated by some researchers (Kortei, Odamtten, Appiah, et al., 2015; Kortei, Odamtten, Obodai, et al., 2015; Kpodo et al., 1996, 2000; Narh et al., 2011) which points to the likelihood of transfer of fungal contaminants from these grains into these local cereal beverages and so good manufacturing and hygiene practices must be observed for the safety of these beverages. The aim of this study, therefore, was to investigate the mycofloral (fungal) and chemical (pH) qualities of the “solom” beverage available for consumption from their respective sales points.

## 2 MATERIALS AND METHODS

### 2.1 Study design and study area

This study was quantitative and purely experimental. It was conducted at the Microbiology Laboratory, School of Allied Health Sciences, University of Health and Allied Sciences, Ho, Volta Region, Ghana.

### 2.2 Sampling

**Figure 1:**
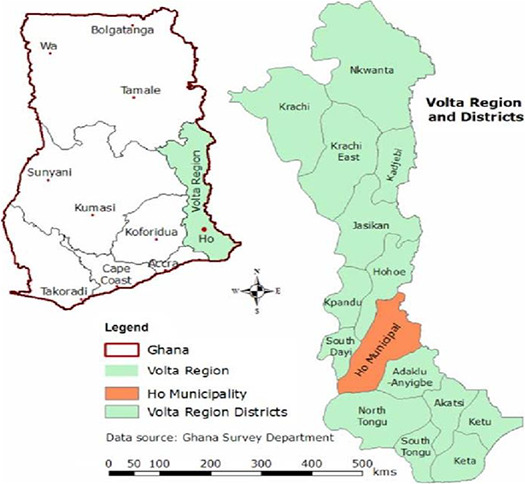
Ho Municipality in the Volta Region of Ghana. Adapted from Arku et al. (2008)

A total of 30 “solom” samples were purchased from ten (10) different locations in Ho, Volta Region, Ghana (Figure 1). From each location, three (3) different samples were purchased. They were stored in sterile plastic bottles and kept in an ice chest immediately after purchase and were transported to the laboratory for analysis.

## 3 FUNGAL ISOLATION

### 3.1 Equipment and materials sterilization

Sterilization of equipment was carried out before and after the analysis to prevent cross‐contamination of samples, and equipment Petri dishes and universal bottles were washed and rinsed very well with soap and under running tap water and kept in a hot air oven for 1 hr at 180°C to ensure sterility. The media were prepared according to manufacturer's guidelines under safety conditions. A naked flame was turned on to prevent microbial contamination during processing sterilization, and 70% alcohol was also used to clean the working bench before and after use.

### 3.2 Fungal plating and incubation

This was carried out according to the procedure outlined for plating by Kortei et al. (2018) with media and process modifications as follows: aliquots of one milliliter (1 ml) of each test sample were added to 9 ml of sterile distilled water and agitated vigorously and was used as the stock solution. The samples were serially diluted 10^–2^ up to 10^–4^ and then plated on Oxytetracycline Glucose Yeast Extract (OGYE) and Dichloran Rose Bengal Chloramphenicol (DRBC) agars. All media were prepared according to the manufacturer's specifications.

It was followed by incubation at 37°C for 7 days. After 1 week, observable molds and yeast appeared for counting and identification.

## 4 FUNGAL IDENTIFICATION AND ENUMERATION

### 4.1 Lacto phenol cotton blue teased mount procedure for identification

A drop of lactophenol cotton blue dye was placed on the slide, and a sterile iron needle was used to transfer a tiny piece of a colony into Lacto Phenol Cotton Blue Dye on the slide. The colony was then teased into very tiny pieces using an iron needle. The slide was covered with a coverslip with a magnification ×400 used. The identification of the fungi was done macroscopically (texture and color in the plate) and microscopically by observation of their cultural and morphological features (Table 1) under the microscope.

**Table 1:** Cultural and morphological characteristics of identified fungi

Fungal specie	Cultural characteristics	Morphological characteristics
*Mucor* spp.	Large white colonies which turns into black later	Erect sporangiophores are formed. Sporangiophore swells at the tip to form sporangia which are globular shaped. Columella is present
*Rhizopus* spp.	White cottony mycelia, with black dots and covers the entire plate	Sporangiospores are produced inside a spherical sporangium. Columella is present on the top of the sporangiophore. Root‐like rhizoids are found
*Penicillium* spp.	Fast‐growing colonies in green color with dense felt conidiophore	Branched conidiophores with chains of conidia looks like a brush
*Aspergillus* spp.	Yellow to green, and black colonies with distinct margin	Conidiophores arise from a footcell. Club‐shaped vesicles at top of the conidiophores. Conidia are found in chains
*Curvularia* spp.	Fast‐growing colonies of suede‐like to downy, brown to blackish	Conidiophores are erect, straight to flexous, septate, often geniculate (producing conidia in sympodial succession)
*Cladosporium* spp.	Colonies are mostly greyish‐olive appearance and later powdery	Conidiophores arising laterally or terminally from the hyphae. Bears chains of conidia
*Fusarium* spp.	White‐pink sparse aerial mycelia becoming felty	Macroconidia sparse, borne on phialides on branched conidiophores (Septate banana‐shaped).
*Rhodotorula* spp.	Soft, smooth, moist and mucoid	Round or oval shaped budding cells

Sources: Samson and van Reenen‐Hoekstra (1988); da Cunha et al. (2013); Madrid et al. (2014); Samson et al. (2000)

Molds and yeast that appeared were identified by their cultural and morphological characteristics using standard identification manuals (Moss, 1989; Samson et al., 2000).

Enumeration was carried out by a colony counter (STAR 8500 Funke Gerber). Colony‐forming unit per milliliter was calculated using the formula,

(1) $$\text{CFU}/\text{ml}=\frac{\text{No}.\;\text{of colonies}\times \text{reciprocal of dilution}}{\text{The volume of the culture plate}}$$

Fungal counts were recorded in standard form and then transformed into the logarithmic form as described by Kortei et al. (2018) and Odamtten et al. (2018).

Percentage occurrence of fungal species was calculated using the formula,

(2) $$\text{Percentage (}\%\text{) occurrence of fungal species}\;=\frac{\text{Number of Species}}{\text{Total number of fungi isolated}}\times 100$$

#### 4.1.1 Determination of pH

The pH of samples was determined directly with a bench pH meter (Jenway 3510) after calibration using standard buffers 4.0 and 7.0 pH.

#### 4.1.2 Data analysis

Procedures of fungal counts and pH were carried out in triplicates, and data collected were subjected to a single‐factor analysis of variance (ANOVA). Differences among means were separated using Duncan's multiple range test (DMRT) and significances were accepted at a 5% level (*p* < .05) using Statistical Package for the Social Sciences (SPSS) software version 22. The analysis was done using the mean counts expressed in the standard forms which were transformed into logarithmic values and results reported as means + standard deviation.

## 5 RESULTS

Results of the different fungal counts of solom from the different locations are represented in Table 2. For OGYE, the fungal counts ranged between 1.68 ± 0.8 and 4.11 ± 0.9 log_10_ CFU/ml for locations H1 and H4, respectively. Counts of solom from the different locations were not statistically significant (*p* > .05). However, H1 and H4 were not comparable (*p* < .05).

**Table 2:** Fungal counts of “solom” enumerated on two (2) different media (OGYE and DRBC) incubated for 5–7 days at 36 ± 1°C

	OGYE		DRBC	
	Fungal counts Log_10_ CFU/ml		Fungal counts Log_10_ CFU/ml	
	Mean + standard deviation	Grand mean + standard deviation	Mean + standard deviation	Grand mean + standard deviation
H1	0.75 ± 1.3	1.68 ± 0.8^c^	1.37 ± 0.3	1.98 ± 0.7^ab^
	2.24 ± 0.4		1.79 ± 0.3	
	2.05 ± 0.3		2.78 ± 1.1	
H2	2.76 ± 0.7	2.82 ± 0.4^ab^	3.72 ± 0.8	2.95 ± 0.9^ab^
	2.40 ± 0.9		3.08 ± 1.0	
	3.28 ± 0.5		2.05 ± 1.5	
H3	3.83 ± 0.5	3.22 ± 0.6^ab^	4.30 ± 1.1	3.52 ± 0.8^ab^
	3.05 ± 0.6		3.55 ± 0.3	
	2.78 ± 1.1		2.72 ± 0.9	
H4	3.80 ± 0.9	4.11 ± 0.9^a^	2.76 ± 0.7	2.92 ± 0.3^ab^
	5.20 ± 1.3		2.72 ± 0.9	
	3.31 ± 0.5		3.28 ± 0.5	
H5	2.52 ± 0.4	3.06 ± 0.7^ab^	3.92 ± 1.1	3.19 ± 0.6^ab^
	3.90 ± 0.9		2.84 ± 0.2	
	2.75 ± 0.9		2.80 ± 0.4	
H6	3.45 ± 1.1	2.69 ± 0.7^ab^	0.75 ± 0.5	1.71 ± 0.8^ab^
	2.38 ± 0.8		2.30 ± 0.4	
	2.25 ± 0.3		2.08 ± 0.4	
H7	1.78 ± 1.1	3.18 ± 1.4^ab^	2.99 ± 0.9	3.11 ± 0.2^ab^
	3.26 ± 1.1		3.35 ± 1.5	
	4.49 ± 0.6		2.98 ± 0.7	
H8	4.30 ± 1.1	3.30 ± 0.9^ab^	2.73 ± 0.7	2.66 ± 0.5^ab^
	2.89 ± 1.0		2.17 ± 0.4	
	2.72 ± 0.9		3.08 ± 0.8	
H9	3.92 ± 1.1	3.19 ± 0.6^ab^	1.94 ± 0.5	2.78 ± 0.8^ab^
	2.84 ± 0.2		2.72 ± 0.9	
	2.80 ± 0.7		3.70 ± 0.8	
H10	3.72 ± 0.8	2.84 ± 0.9^ab^	2.57 ± 0.9	3.09 ± 0.7^ab^
	3.01 ± 1.0		3.87 ± 0.8	
	1.80 ± 1.4		2.81 ± 0.4	

Note

Means in a column with the same superscript letters are not statistically different (*p* > .05).

For DRBC, the counts were in the range of 1.71 ± 0.8–3.52 ± 0.8 log_10_ CFU/ml for locations H6 and H3, respectively. Statistically, the counts were all comparable (*p* > .05). There was an observed low fungal count generally. The range of counts was within the acceptable to borderline range of microbiological counts for ready‐to‐eat foods as prescribed by the International Commission for Microbiological Specification of Foods (ICMSF, 1998) (Table 3).

**Table 3:** Guidance on the interpretation of results for specific foodborne pathogens in ready‐to‐eat food in general (colony‐forming unit (CFU/g)

Hazard	Result (CFU/g)	Interpretation	Likely cause
Fungi	<10^2^ or 2 log_10_	Satisfactory	
	10^2^–<10^4^ 2–4 log_10_	Marginal/borderline	Process controls not fully achieved or possible raw material contamination
	>10^4^ 4 log _10_	Unsatisfactory (potentially injurious to health and/or unfit for human consumption)	Inadequate time and temperature control during cooling and subsequent storage allowing spores to germinate and multiply

Note

International Commission for Microbiological Specifications for Food (ICMSF, 1998).

The pH values were in the range of 3.03 ± 0.09–4.03 ± 0.23, respectively, for HS6 and HS10. Statistically, there were no significant differences (*p* > .05) observed among the pH values of the solom from the respective locations. The trend of pH of the samples collected from the Ho municipality showed no particular trend (Table 4).

**Table 4:** pH values of the “solom” samples obtained from the various locations in Ho

Location	pH	Mean ± standard deviation
HS1	3.42	3.42 ± 0.02^a^
	3.38	
	3.40	
HS2	3.24	3.26 ± 0.02^a^
	3.26	
	3.28	
HS3	3.22	3.19 ± 0.03^a^
	3.18	
	3.17	
HS4	3.48	3.60 ± 0.10^a^
	3.67	
	3.65	
HS5	3.11	3.15 ± 0.03^a^
	3.16	
	3.17	
HS6	3.14	3.03 ± 0.09^a^
	2.97	
	2.99	
HS7	3.84	3.84 ± 0.16^a^
	3.69	
	4.00	
HS8	3.00	3.10 ± 0.11^a^
	3.21	
	3.09	
HS9	3.77	3.71 ± 0.05^a^
	3.67	
	3.69	
HS10	3.78	4.03 ± 0.23^a^
	4.21	
	4.11	

Note

Means in a column with the same superscript letters are not statistically different (*p* > .05).

**Plate 2:**
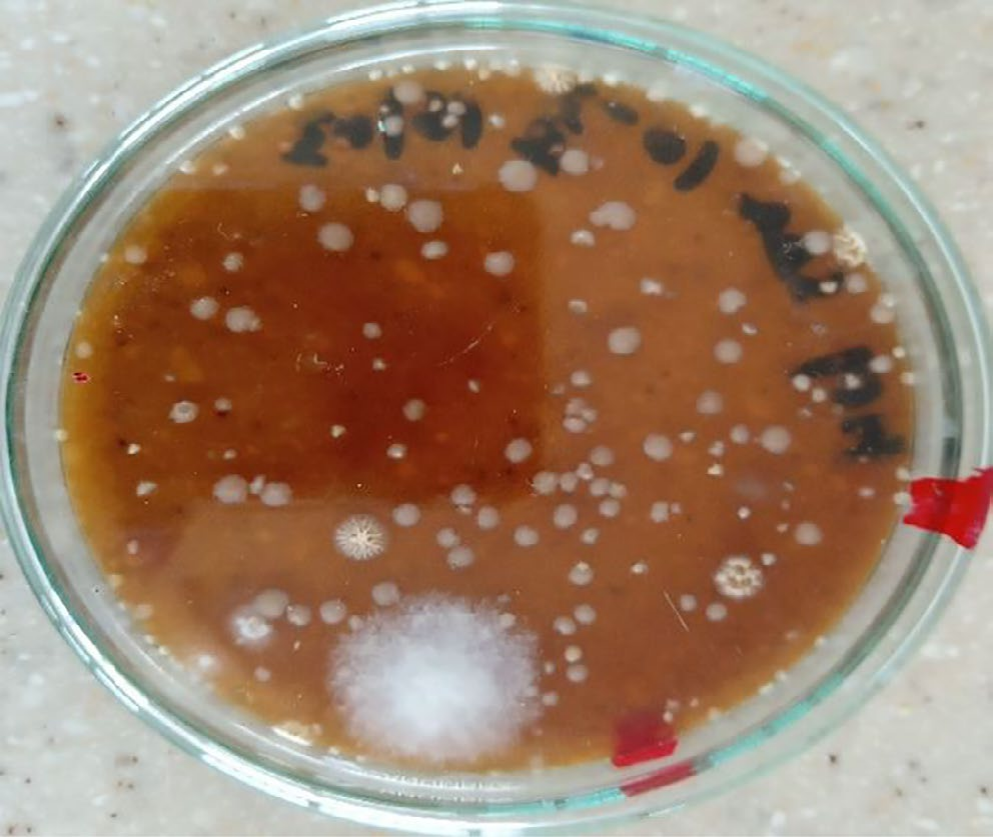
Macroscopic view of *Fusarium oxysporum*, *Rhodoturula* spp. isolated from “solom”

**Plate 3:**
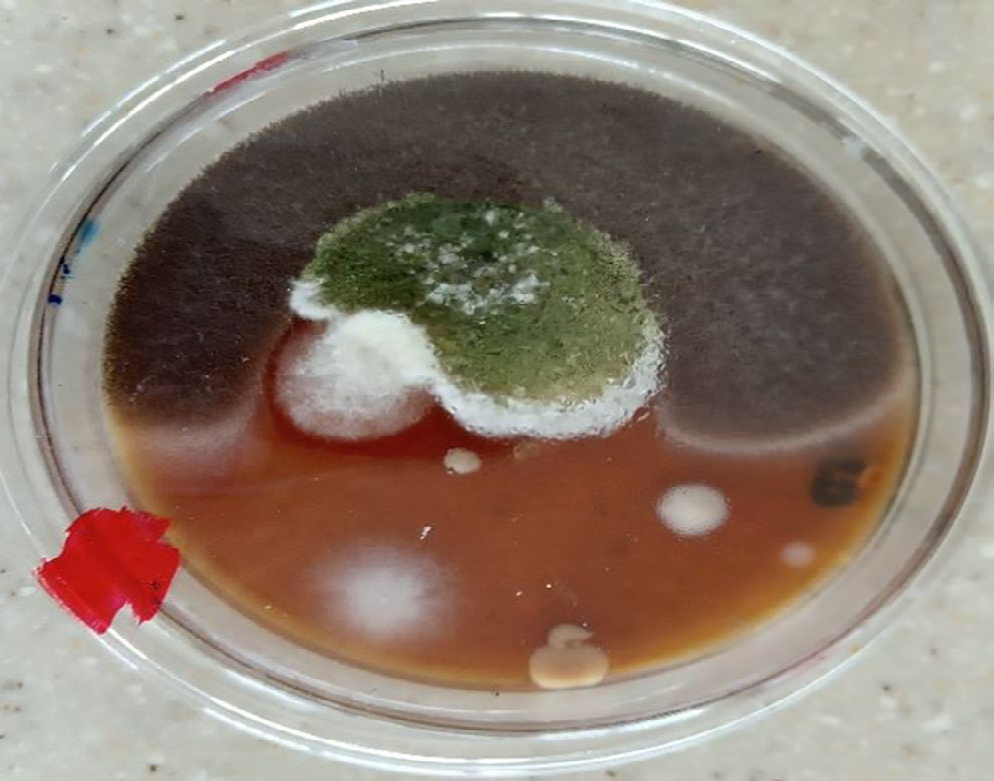
Macroscopic view of *Aspergillus* spp., *Fusarium* spp., *Rhodotorulla* spp., *Penicillium* spp. isolated from “solom”

**Plate 4:**
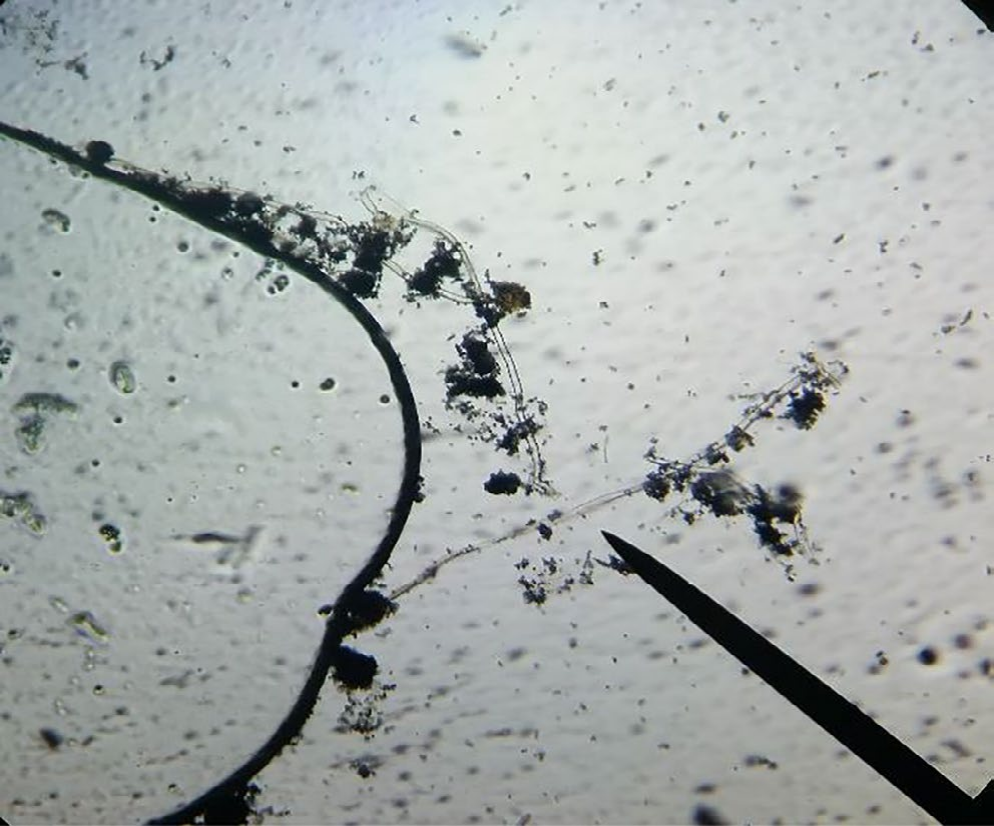
Microscopic view of *Aspergillus* spp. isolated from “solom”

Table 5 summarizes the fungal species isolated in solom obtained from the different locations in the Ho Municipality. A total of fourteen (14) fungal species belonging to eight (8) fungal genera were isolated on both OGYE and DRBC media; *Aspergillus (A. niger, A. flavus, A. fumigatus, A. parasiticus, A. alutaceaus, A. terreus), Rhizopus (R. stolonifer), Mucor (M. racemosus), Fusarium (F. oxysporum), Penicillium (P. digitatum, P. verucosum), Cladosporium (C. cladosporoides), Curvularia (C. lunata),* and *Rhodotorula* sp. were recorded (Plates 2, 3, 4).

**Table 5:** Fungal species and their percentage (%) occurrence in “solom” from various locations in Ho cultured on two (2) different media (OGYE and DRBC) and incubated for 5–7 days at 36 ± 1°C

	OGYE		DRBC	
	Species identified	Percentage (%) occurrence	Species identified	Percentage (%) occurrence
H1	*Aspergillus niger*	25	*Aspergillus flavus*	15
	*Aspergillus fumigatus*	75	*Aspergillus niger*	25
			*Aspergillus fumigatus*	60
H2	*Fusarium oxysporum*	85	*Rhizopus stolonifera*	10
	*Aspergillus fumigatus*	15	*Rhodotorula* spp.	90
H3	*Penicillium digitatum*	16	*Aspergillus niger*	44
	*Aspergillus flavus*	24	*Rhodotorula* spp.	40
	*Aspergillus niger*	35	*Penicillium* spp.	16
	*Aspergillus fumigatus*	15		
H4	*Fusarium oxysporum*	30	*Fusarium oxysporum*	80
	*Aspergillus niger*	40	*Rhodotorula* spp.	20
	*Aspergillus flavus*	30		
H5	*Mucor racemosus*	75	*Aspergillus fumigatus*	100
	*Aspergillus fumigatus*	25		
H6	*Penicillium verucosum*	65	*Rhizopus stolonifer*	45
	*Aspergillus flavus*	35	*Curvularia lunata*	20
			*A. niger*	35
H7	*Aspergillus niger*	5	*Cladosporium cladosporiodes,*	30
	*Mucor racemosus*	75	*A. flavus*	36
	*Aspergillus fumigatus*	20		
			*Penicilium digitatum*	34
H8	*Fusarium oxysporum*	50	*Aspergillus niger*	35
	*Penicillium digitatum*	25	*Fusarium oxysporum*	30
	*Aspergillus parasiticus*	25	*Aspergillus terreus*	35
H9	*Curvularia lunata*	45	*Fusarium oxysporum*	98
	*Aspergillus alutaceous*	55	*Aspergillus flavus*	2
H10	*Fusarium oxysporum*	64	*Fusarium oxsporum*	45
	*Rhodotorula* spp.	36	*Aspergillus niger*	26
			*Penicillium digitatum*	29

The species isolated from the various locations were *Aspergillus flavus, Aspergillus niger Aspergillus fumigatus* (H1)*, Rhizopus stolonifera, Rhodotorula* spp. *Fusarium oxysporum, Aspergillus fumigatus* (H2)*, Aspergillus niger, Rhodotorula* spp., *Penicillium digitatum, Aspergillus flavus, Aspergillus fumigatus* (H3)*, Fusarium oxysporum, Rhodotorula* spp., *Aspergillus niger, Aspergillus flavus* (H4)*, Mucor racemosus, Aspergillus fumigatus* (H5)*, Penicillium verucosum, Aspergillus flavus, Rhizopus stolonifera, Curvularia lunata, Aspergillus niger* (H6)*, Aspergillus niger, Mucor racemosus, Aspergillus fumigatus, Cladosporium cladosporiodes, A. flavus, Penicillium digitatum* (H7)*, Fusarium oxysporum, Penicillium digitatum, Aspergillus parasiticus, Aspergillus niger, Fusarium oxysporum, Aspergillus terreus* (H8)*, Curvularia lunata, Aspergillus alutaceous, Fusarium oxysporum, Aspergillus flavus* (H9), and *Fusarium oxysporum, Rhodotorula* spp., *Aspergillus niger, and Penicillium digitatum* (H10).

## 6 DISCUSSION

### 6.1 pH

Cereals are susceptible to infections by a broad continuum of plant pathogens. The globalization of cereal commerce is largely linked to increased fungal infection and cross‐contamination hazards (Waage et al., 2006). Fungal growth is influenced by favorable environmental conditions such as pH, temperature, moisture, and light. The pH of a medium is positively correlated with the growth of most fungi. All samples analyzed were acidic (pH 3.03 ± 0.09–4.03 ± 0.23) as referenced from the pH scale. The range of acidity in cereal beverages has been observed by some researchers namely Akoma et al. (2014) and Popoola (2019) who reported pH ranges of 3.91–3.96 and 3.13–3.36 as appropriate for fungal growth in samples of “akamu” and “kununzaki,” respectively, prepared from different cereals in Nigeria and ascribed the acidity to the presence of lactic acid bacteria during the fermentation process. Lactic acid bacteria (LAB) represent an pervasive and assorted species with common feature of lactic acid production as a result of sugar metabolism from the cereal which leads to an acidification of the environment down to a pH of around 3.5 (Charlier et al., 2009) which aids in the preservation of the food. Presumably, the drinks of lower pH had been stored over a longer period of time which is likely to eliminate most fungi thus safer for consumption.

It is noteworthy the range of pH values recorded in this work were lower than pH ranges of 4.6–6.8 and 4.5–7.0 described as optimum for the growth of fungi by Jackson et al. (1991) and Weyman‐Kaczmarkowa and Pędziwilk (2000), respectively. Likewise, Anupma and Tamang (2020) recorded an average of pH 5.3. Nonetheless, a much higher pH range was reported by Yamanaka (2003) as 7–9 for optimum growth of fungi. The acidity of this beverage tended to be directly proportional to the storage period (increase with an increase in the fermentation period) resulting in spoilage. Consequently, the low pH values may have inhibited the growth of some fungal species isolated.

### 6.2 Fungal counts

Fungal counts observed in this work were in the same range of values of 2.098–4.23 log_10_ CFU/ml reported by Aboagye et al. (2020) as counts of “asaana” a beverage of maize from Ghana. Minamor, Mensah, et al. (2017) recorded fungal counts of <10^4^ CFU/ml in “pito” a cereal beverage of sorghum were found to be within the permissible limits.

Contrarily, Oriola et al. (2017) reported higher fungal counts of range 3.4 × 10^5^ ± 0.10–4.5 × 10^6^ ± 0.10 (5.53–6.65 log_10_) CFU/ml from “Otika” a Nigerian cereal beverage of sorghum. In a related study, Anupma and Tamang (2020) recorded an average fungal population of 4.9 × 10^5^ (5.69 log_10_) CFU/g from amylase and alcohol‐producing starters in India. From Nigeria, Popoola (2019) reported a range between 1.30 × 10^5^ CFU/ml and 1.74 × 10^5^ (5.11–5.23 log_10_) CFU/ml in “akamu” samples obtained from different cereals. High colony counts >10^4^ are an indication of spoilage as a result of either poor hygiene or poor quality of cereals and water used in the preparation of the beverage.

Mossel et al. (1986) highlighted that variation in fungal counts could be attributed to differences in compliance with Good Manufacturing Practices (GMP) conditions during the growing, processing, or storage of the raw material of the food. Furthermore, the effect of storage on the viability of the fungal propagules could also be worth considering. Lastly, the high incidence of a particular fungal specie may indicate the presence of mycotoxins. A consumer must be made aware the consequences of drinking “solom” not hygienically prepared and also stored for longer periods as may contain greater fungal counts; hence, its safety is doubtful.

The range of counts recorded in this work were, however, within the acceptable range of microbiological counts for ready‐to‐eat foods as prescribed by the International Commission for Microbiological Specification of Foods (ICMSF, 1998).

### 6.3 Fungal species

Copious types of microorganisms, molds, bacteria, and yeasts are established in the naturally fermented starters. Our findings from this work compare favorably well with results by Fadahunsi et al. (2013) and they also identified the fungi *Saccharomyces cerevisiae*, *Candida krusei,* and *Aspergillus niger* in the both fresh and stored samples of burukutu and pito. The genera *Aspergillus, Fusarium,* and *Penicillium* are often associated with contamination of agricultural products from the field, during storage and transportation. Fungal contamination on grain during storage and transportation occurs frequently in the intercontinental trade of cereals. Wheat, barley, com, and other cereals are regulated for their mold (physical) and mycotoxin contaminations by the quarantine service of export and import harbors.

Misihairabgwi et al. (2018) identified some mycotoxins suggestive of the presence of fungi of the genera *Aspergillus, Fusarium, Penicillium, Alternaria,* and *Claviceps* from “Oshikundu” a beverage produced from millet in Namibia. From Malawi, Matumba et al. (2011) detected aflatoxins in 100% of the sorghum malt samples used for the production of the traditional opaque beverage “tobwa” used for producing the traditional sorghum opaque beer. This points to the presence of *Aspergillus* spp. in the sorghum samples.

Although mycotoxins were not investigated for in this study, several reports exist on the occurrence of some kinds of mycotoxins of public health interests viz‐viz their toxicogenic fungi.

In a related study, Aboagye et al. (2020) recorded a total of 11 fungal species belonging to 4 genera were isolated, and namely *Aspergillus, Fusarium, Penicillium, and Rhodotorulla* spp from “asaana” (corn malted drink) from Ghana. From Nigeria and some parts of Benin, fungal species belonging to the genera *Aspergillus* and *Penicillium* have been reported to be associated with “Ogi” (Phiri et al., 2020).

Fungi isolated by Elmahmood and Doughari (2007) included *Penicillium digitatum, Aspergillus fumigatus, Rhizopus nigricans, and Mucor sitophila* from “Kununzaaki” (cereal beverage from Nigeria). The incidence of these fungal species has been linked to the spoilage of beverages as explained by Kolawole et al. (2007). Anupma and Tamang (2020) also isolated filamentous molds belonging to seven genera, *Mucor, Aspergillus, Penicillium, Bjerkandera, Rhizopus, Trametes,* and *Cladosporium* from amylase and alcohol‐producing starters in India.

Conversely, a study carried out in Burkina Faso, Bationo et al. (2015) reported an insignificant incidence of *Aspergillus* in sorghum malt samples. Similarly, insignificant *Aspergillus* were detected in the beverage “dolo” prepared from sorghum malt samples from the same study. Minamor et al. (2017) also isolated *Saccharomyces cerevisiae*, as the only fungus associated with fermentation of “pito” in Accra, Ghana.

Lee and Lee (2002) explained fungi and lactic acid bacteria grow simultaneously in cereal beverages and play important roles in the later stage of alcoholic fermentation, and the mixed culture also contributes to some sensorial properties of the beverage such as the more intense flavor of some beverages. Molds and other microorganisms convert unpalatable carbohydrates of low digestibility and proteins into palatable sugars and amino acids with a high conversion efficiency.

The occurrence of toxigenic fungi in these cereals and beverages is suggestive of the potential presence of their mycotoxins. Mycotoxins are secondary metabolites of filamentous fungi which are mostly toxic, carcinogenic, mutagenic, and teratogenic even at the minutest of concentrations (Ezekiel et al., 2018; Omotayo et al., 2019).

Some mycotoxins including aflatoxins, deoxynivalenol (DON), fumonisins, ochratoxins, and zearalenone (ZEN) have previously been reported in cereal beverages from Africa and Europe (Abia et al., 2013; Bertuzzi et al., 2011; Ezekiel et al., 2018).

Although not investigated in this present study, incidences of different mycotoxins in traditional as well as nontraditional cereal beverages have been reported in the literature. Of particular concern to food safety and public health are those of the toxicogenic fungi belonging to the genera; *Fusarium* (zearalenone, fumonisins, deoxynivalenol (DON), ergot alkaloids), *Aspergillus* (aflatoxins, ochratoxins, ergot alkaloids), and *Penicillium* (ochratoxins, ergot alkaloids, patulin, cyclopiazonic acid). Abia et al. (2013) also revealed the occurrence of DON (93%) in locally brewed maize beer from Cameroon. Similarly, Ayalew et al. (2006) found high 249 contamination of sorghum with DON (range: 50–2,340 μg/kg, mean: 360 μg/kg), the main cereal 250 used for production of “burukutu” and “pito.” Ezekiel et al. (2018) also reported fumonisin contamination of levels of 170 μg/kg and 2.9 μg/kg, respectively, for “kununzaki” and “pito.” Similarly, they observed levels of 0.2 μg/kg for both cereal beverages. Abia et al. (2013) again reported 645 μg/kg of aflatoxins in “sha” a local beer produced from maize in Cameroun. Likewise, Atter et al. (2015) also recorded aflatoxin levels of range 0.51–1.63 μg/kg in “ice‐kenkey” (a beverage of maize from Ghana).

According to WHO (2018), some mycotoxins such as fumonisins have been linked to esophageal, liver, and several types of cancer and perhaps spinal defects in neonates in Sub‐Saharan Africa and China, while zearalenone has gained notoriety in causing fluctuations in the estrogen levels of humans resulting in reproductive disorders. The presence of zearalenone in large quantities can cause disruption in conception, abortion, and other problems.

Kwashiorkor in children is aggravated by long‐term exposure to aflatoxin. Commonly, aflatoxins cause jaundice, hepatomegaly, splenomegaly, tachycardia, and anorexia beside others. Ochratoxins are also well known for causing damage to the liver (Nyanzi & Jooste, 2012).

It is noteworthy in many illustrations, assigning a favored basis of contamination can be thought‐provoking. Moreover, the environment of a specific manufacturing plant can be highly variable and unique, such that it may be necessary to clarify the specific sources of contamination in each particular situation (Garijo et al., 2015; Hernández et al., 2018). Microbial contamination throughout the market chain is an inevitable occurrence.

The presence of pathogenic microorganisms and mycotoxins in beverages constitutes a Public Health hazard for consumers and economic loss for the producers (Granados‐Chinchilla et al., 2018) as many of these fungi are in the spoilage category and the presence of toxigenic fungi is indicators of a possible mycotoxin contamination risk (Anjorin et al., 2013). It is of paramount importance that these fermented products be prepared under good sanitary and hygienic conditions including application. To mitigate the effects of the natural presence of microbes in the food chain, the application of Good Hygiene Practices (GHP) and Good Manufacturing Practices (GMP) allows a minimization in the contamination to satisfactory levels.

## 7 CONCLUSION

Our findings on fungal diversity in Solom from the Ho Municipality of Ghana may supplement the microbial miscellany in ecosystems of Ghana, which is one of the potential biodiversity hot spots of the world. We identified eight (8) genera with 14 species of fungi represented by *Aspergillus (A. niger, A. flavus, A. fumigatus, A. parasiticus, A. alutaceaus, A. terreus), Rhizopus (R. stolonifer), Mucor (M. racemosus), Fusarium (F. oxysporum), Penicillium (P. digitatum, P. verucosum), Cladosporium (C. cladosporoides), Curvularia (C. lunata),* and *Rhodotorula* sp. as fungal contaminants of the millet beverage solom. Fungal species present in this traditional cereal‐based beverage (“solom”) were morphologically, ecologically, and showed high diversity within the community. The pH of the cereal beverage (solom) was found to be between the ranges of 3.03 ± 0.09–4.03 ± 0.23 which suggested acidity.

## CONFLICT OF INTEREST

The authors declare that they have no conflicts of interest.

## Supporting information

Supplementary Material

## Data Availability

The data that support the findings of this study are available from the corresponding author upon reasonable request.
