# Supplementary material for: Fungal diversity of “solom” a Ghanaian traditional beverage of millet (Pennisetum glaucum)
Source: Food Sci Nutr. 2020 Nov 29;9(2):811–21. doi: 10.1002/fsn3.2045 (PMC7866583; doi:10.1002/fsn3.2045)
Supplement: Supplementary file 1 — Supplementary Material [file FSN3-9-811-s001.docx]

Supplementary Information


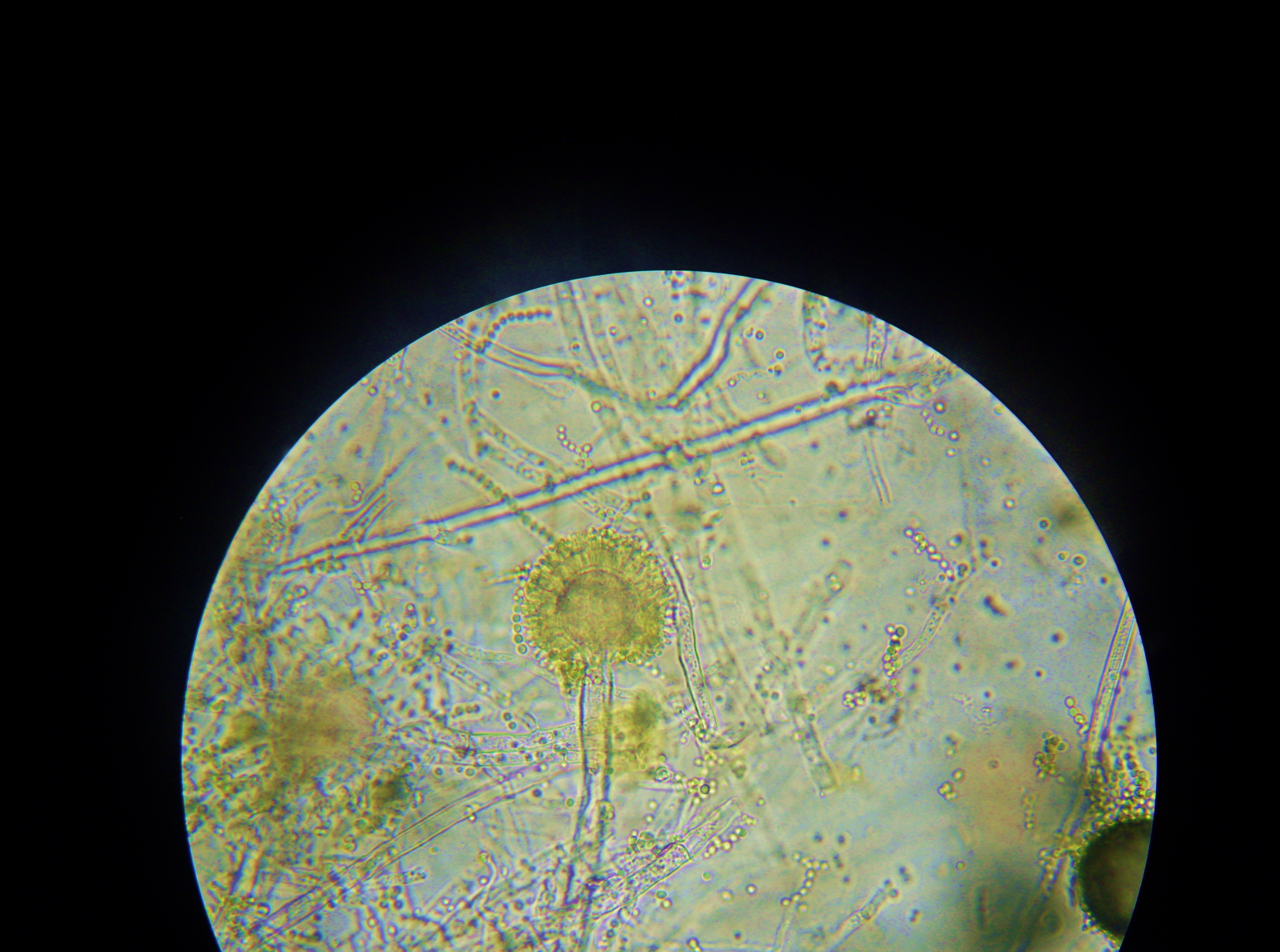


*Aspergillus flavus mag.x400*


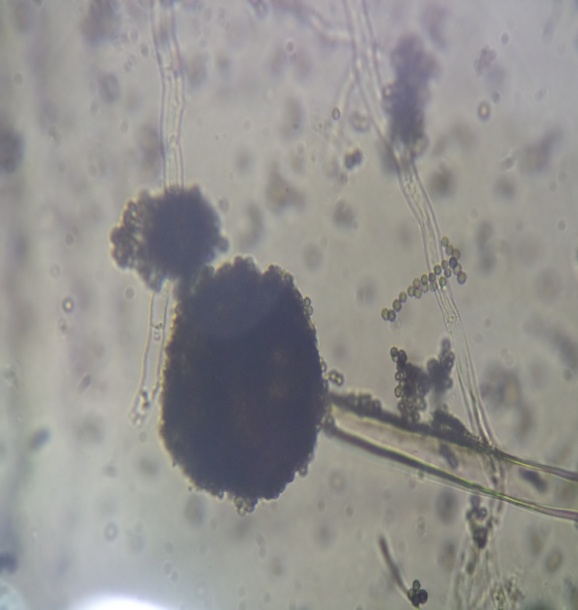


*Aspergillus niger mag.x400*


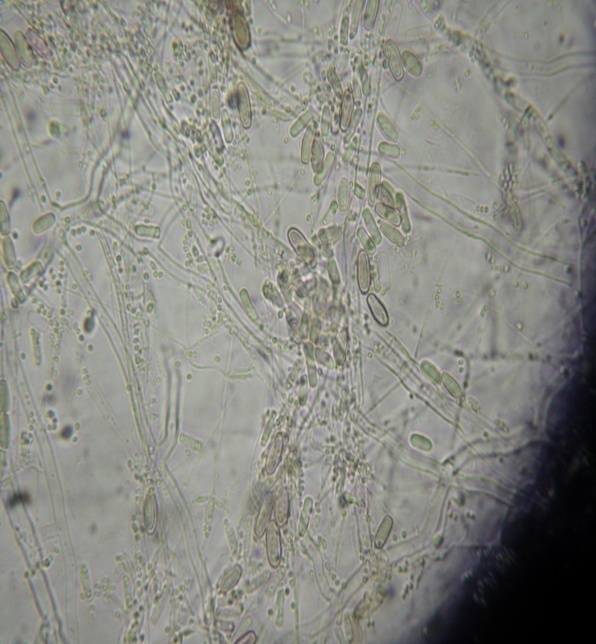


*Fusarium oxysporum mag.x400*


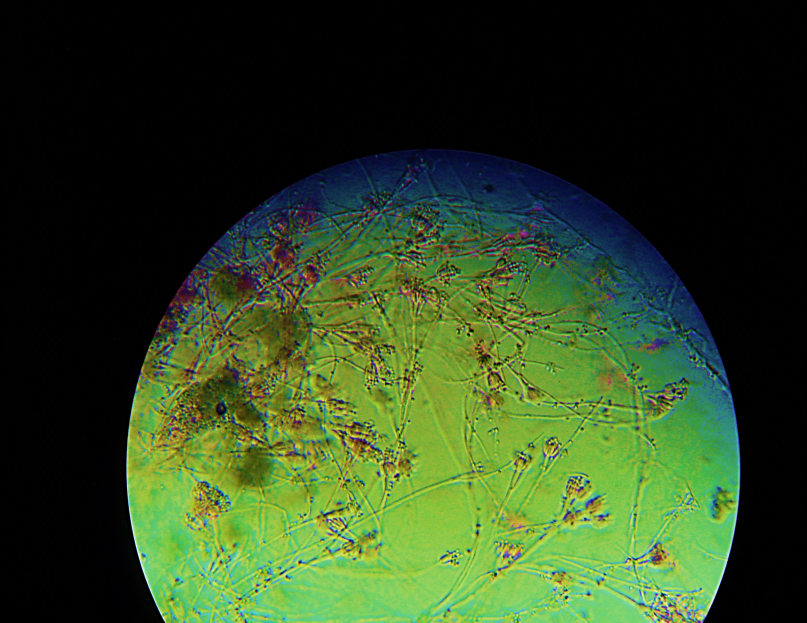


*Penicillium digitatum mag.x400*
